# Decoding tumor-fibrosis interplay: mechanisms, impact on progression, and innovative therapeutic strategies

**DOI:** 10.3389/fphar.2024.1491400

**Published:** 2024-10-23

**Authors:** Huiguang Chen, Xuexin Xu, Jingxian Li, Yu Xue, Xin Li, Kaiyu Zhang, Haihui Jiang, Xiaoliu Liu, Mingzhe Li

**Affiliations:** ^1^ Institute of Infection, Immunology, and Tumor Microenvironment, School of Medicine, Wuhan University of Science and Technology, Wuhan, China; ^2^ Department of Anatomy, School of Medicine, Wuhan University of Science and Technology, Wuhan, China

**Keywords:** fibrosis, tumor, CAFs, TGF-β, EMT

## Abstract

Malignant tumors are a category of diseases that possess invasive and metastatic capabilities, with global incidence and mortality rates remaining high. In recent years, the pivotal role of fibrosis in tumor progression, drug resistance, and immune evasion has increasingly been acknowledged. Fibrosis enhances the proliferation, migration, and invasion of tumor cells by modifying the composition and structure of the extracellular matrix, thereby offering protection for immune evasion by tumor cells. The activation of cancer-associated fibroblasts (CAFs) plays a significant role in this process, as they further exacerbate the malignant traits of tumors by secreting a variety of cytokines and growth factors. Anti-fibrotic tumor treatment strategies, including the use of anti-fibrotic drugs and inhibition of fibrosis-related signaling pathways such as Transforming Growth Factor-β (TGF-β), have demonstrated potential in delaying tumor progression and improving the effectiveness of chemotherapy, targeted therapy, and immunotherapy. In the future, by developing novel drugs that target the fibrotic microenvironment, new therapeutic options may be available for patients with various refractory tumors.

## 1 Introduction

Malignant tumors are a category of abnormal cellular proliferation diseases characterized by invasiveness and metastatic potential. In 2022, it was estimated that there were 20 million new cases and 9.7 million deaths worldwide (Bray et al., 2024). Although cancer treatment methods, such as surgery, radiotherapy, chemotherapy, targeted therapy, and immunotherapy, have continuously advanced, the complexity and heterogeneity of the disease make it challenging to cure, posing a significant global public health problem (Hirsch et al., 2017). Hanahan stated that the progression of tumors involves more than just an increase in tumor cell numbers and must be understood within the framework of the “tumor microenvironment (TME).” (Hanahan and Weinberg, 2011) TME is a complex system composed of tumor cells, stromal cells, immune cells, blood vessels, and the extracellular matrix (ECM). A growing body of research indicates that the TME is vital in the growth, invasion, metastasis, and treatment resistance of multiple tumors. Additionally, tumor cells can secrete cytokines and growth factors, inducing stromal cell reprogramming to regulate the TME, providing new perspectives for clinical treatment (Ding et al., 2024; Mao et al., 2024; Nie et al., 2024).

Fibrosis is defined as the excessive accumulation of ECM components, like collagen, resulting in abnormal alterations in tissue structure and function. It is a chronic and progressive process, typically linked to prolonged inflammation and damage (Rimal et al., 2022). Additionally, fibrosis, as an essential part of the TME, is mainly manifested by the excessive deposition of ECM and the abnormal activation of stromal cells, including tumor-associated fibroblasts (CAFs) and myofibroblasts (Rimal et al., 2022). With deeper research, the intricate interactions between fibrosis and tumors are increasingly being clarified. Fibrosis facilitates tumor cell proliferation, invasion, and immune evasion (Thomas and Radhakrishnan, 2019; Metcalf et al., 2022). Additionally, tumor cells further aggravate fibrosis by secreting pro-fibrotic factors and inducing chronic inflammatory responses (Wu et al., 2020; Giarratana et al., 2024). Anti-fibrotic treatments, including anti-TGF-β therapy and targeting CAFs, have demonstrated important potential in the treatment of malignant tumors (Mohapatra et al., 2022; Li J. et al., 2023). Thus, comprehending the interaction mechanisms between fibrosis and different tumors is crucial for further research, developing novel therapeutic strategies, and enhancing cancer treatment efficacy.

## 2 Mechanisms that promote fibrosis in malignant tumors

### 2.1 The function of CAFs

#### 2.1.1 Pro-fibrotic factors induce the activation of CAFs

Pro-fibrotic factors are vital in the activation and transformation of CAFs. Malignant tumor cells secrete pro-fibrotic factors (like TGF-β and PDGF), which can directly induce the transformation of fibroblasts into CAFs. CAFs represent one of the main cell types within the TME. The high expression of α-smooth muscle actin (α-SMA) and the biological characteristics of secreting multiple cytokines by CAFs play a vital role in tumor fibrosis (Geng et al., 2021). In renal clear cell carcinoma (RCC), cancer cells secrete TGF-β, which induces the transformation of normal fibroblasts into CAFs through the TGF-β-Smad2/3 pathway (Wang Y. et al., 2024). circ_0020256 is highly expressed in cholangiocarcinoma (CCA) and enhances CCA cells’ secretion of TGF-β1, which subsequently activates CAFs via Smad2/3 phosphorylation. Mechanistically, circ_0020256 stabilizes KLF4 mRNA by recruiting EIF4A3 protein and increasing its expression. KLF4 then binds to the TGF-β1 promoter, enhancing its transcription in CCA cells (Li Z. et al., 2023). Hepatocellular carcinoma (HCC) cells secrete exosomes containing miRNA-21, directly targeting the PTEN gene and activating the PDK1/AKT signaling pathway, which promotes the transformation of normal hepatic stellate cells (HSCs) into CAFs with pro-cancer characteristics (Zhou et al., 2018). In oral squamous cell carcinoma (OSCC), PDGF secreted by cancer cells binds to PDGFR-β, activating lncRNA LURAP1L-AS1, which subsequently regulates the IKK/NF-κB signaling pathway, facilitating the activation and transformation of fibroblasts (Ren et al., 2021).

#### 2.1.2 ECM remodeling

CAFs contribute to malignant tumor fibrosis by enhancing the synthesis of collagen, fibronectin, and other ECM components, resulting in excessive ECM accumulation in tissues. CAFs produce and secrete substantial quantities of type I and III collagen, the primary components of the ECM. The over-deposition of these collagens results in tissue stiffening and densification (Xu et al., 2024). Gastric cancer cells induce the abnormal expression and secretion of collagen by activating the FAK/AKT pathway in CAFs, driving malignant transformation and fibrosis (Zhang J. et al., 2024). CAFs secrete small extracellular vesicles (sEVs) that associate with the ECM; these sEVs are enriched with active lysyl oxidase (LOX). LOX interacts with collagen I under the action of sEVs, facilitating collagen cross-linking. Moreover, integrin α2β1 in sEVs mediates their binding to collagen, further strengthening the cross-linking process (Liu Y. et al., 2023). In lung cancer models, increased lipid droplet (LD) content in CAFs promotes their pro-tumor phenotype, characterized by high expression of α-SMA and collagen α-2 chain (COL1A2) (Zhang et al., 2022).

Moreover, CAFs secrete matrix metalloproteinases (MMPs) and tissue inhibitors of metalloproteinases (TIMPs), which control the degradation and remodeling of the ECM. In the process of fibrosis, CAFs aggravate fibrosis by adjusting the balance between MMPs and TIMPs, inhibiting normal ECM degradation, and facilitating ECM accumulation and stabilization (Najafi et al., 2019). For instance, when co-cultured with gastric cancer cells, CAFs significantly upregulate IL-17a expression and enhance the expression of MMP2 and MMP9, while downregulating their inhibitors TIMP1 and TIMP2 (Zhang J. et al., 2020).

### 2.2 Inflammation

Tumor cells promote fibrosis by persistently releasing inflammatory factors like IL-1, TGF-β1, TNF, and IL-6, which activate the NF-κB and JAK/STAT signaling pathways, inducing fibroblast differentiation into a pro-inflammatory phenotype or myofibroblasts. These fibroblasts further drive fibrosis (Anderson-Crannage et al., 2023). In a lung cancer mouse model, tumor cells induce an inflammatory response in the kidneys by secreting nephrotoxic proteins, which increase the expression of IL-6 and monocyte chemoattractant protein-1 (MCP-1), resulting in glomerular capillary collapse and tumor antigen deposition. Concurrently, the TGF-β signaling pathway is activated, triggering renal fibrosis (Hung et al., 2020). In pancreatic ductal adenocarcinoma (PDAC), tumor cells induce an inflammatory response in pancreatic stellate cells (PSCs) by absorbing lipids, which subsequently promotes PSC activation and triggers fibrosis (Hata et al., 2017). In pancreatic neuroendocrine tumors, cancer cells secrete interleukin-1 (IL-1), which induces CAFs to secrete stromal cell-derived factor 1 (SDF1), aggravating the extent of tumor fibrosis (Lai et al., 2024).

### 2.3 Signaling pathway

#### 2.3.1 TGF-β/Smad signal transduction pathway

The TGF-β/Smad pathway serves as a key regulator in the fibrosis of malignant tumors. This pathway drives fibrosis formation and progression by modulating fibroblast proliferation, differentiation, activation, and the synthesis and deposition of ECM. TGF-β binds to the type II TGF-β receptor (TGF-βRII) on the cell surface, which then activates the kinase activity of TGF-βRI and triggers the phosphorylation of downstream Smad proteins. The phosphorylated Smad2 and Smad3 associate with the co-transcription factor Smad4, forming an active complex that moves into the nucleus to regulate the transcription of fibrosis-related genes (Lee and Massagué, 2022; Li J. et al., 2024).

Activation of the TGF-β-Smad2/3 pathway induces the expression of fibrotic factors, which leads to fibroblast activation, promoting their proliferation and differentiation into myofibroblasts. Myofibroblasts display increased α-SMA expression and an enhanced ability to synthesize ECM, thereby intensifying fibrosis within tumors and promoting tumor growth and progression (Su et al., 2020). The activation of the upstream Notch signaling pathway triggers the TGF-β/Smad pathway, which promotes the migration of mesenchymal stem cells to the stroma and their differentiation into fibroblasts (Peng et al., 2014). In breast cancer, a lack of glutamine can trigger the activation of TGF-β signaling, leading to the activation of associated fibroblasts and subsequent fibrosis. The activity of histone deacetylase 1 and the inhibition of mTORC1 are required for TGF-β signaling activation and the conversion of CAFs into a myofibroblast state (Mezawa et al., 2023). During cachexia, inflammation within tumors drives fibrosis. This process might be driven by TGF-β-induced differentiation of fibroblasts into myofibroblasts, resulting in imbalanced inflammatory cytokine expression, enhanced angiogenesis, and increased ECM components (Lima et al., 2019).

The TGF-β/Smad signaling pathway is essential in fibrosis, tumor progression, and metastasis by enhancing ECM synthesis and deposition. The epigenetic regulators UBR7 and histone methyltransferase EZH2 regulate TGF-β/Smad signaling. With the activation of the TGF-β/Smad pathway, collagen content and lysyl oxidase activity rise, directly impacting ECM stiffness (Adhikari et al., 2024). In unilateral breast cancer-associated lymphedema, TGF-β1 intensifies the fibrosis process by increasing the stiffness of fibroblasts, lymphatic endothelial cells, and lymphatic smooth muscle cells, and by enhancing ECM deposition (Baik et al., 2022).

#### 2.3.2 JAK/STAT signal transduction pathway

The JAK/STAT signaling pathway drives fibrosis formation and reshapes the tumor microenvironment by mediating cell proliferation, differentiation, immune regulation, and inflammatory responses. Cytokines (like IL-6, IFN, and IL-13) bind to receptors, leading to JAK activation, followed by STAT protein phosphorylation. Phosphorylated STAT proteins dimerize and move into the nucleus, where they bind to DNA sequences to regulate the transcription of fibrosis-related genes (Liu X. et al., 2023). Bioinformatics analysis has shown that hub genes are significantly enriched in the JAK/STAT pathway in expression profiles associated with liver fibrosis and liver cancer (Hamdy et al., 2023). With the marked activation of pSTAT5 and pSTAT3, levels of pro-inflammatory and pro-tumor mediators rise, resulting in higher liver tumor burden and significantly increased fibrosis in mice (Cabrera-Galván et al., 2023). In RCC, RCC-derived CXCL5 promotes fibrosis by activating the JAK/STAT3 pathway, facilitating the transformation of normal fibroblasts into CAFs (Liu Y. et al., 2023). Research indicates that reprogrammed mouse liver cells, driven by IL6/Jak/Stat3 signaling pathways, convert into LGR5-positive cells. When transplanted into syngeneic mice, these LGR5-positive cells develop into invasive and metastatic tumors with marked fibrosis, underscoring the significance of the JAK/STAT pathway in malignant tumor fibrosis (Chaker et al., 2024). Research by Grohmann et al. shows that inhibiting STAT-1 signaling prevents T cell recruitment and fibrosis but does not prevent hepatocellular carcinoma; whereas correcting STAT-3 signaling can prevent liver cancer without affecting fibrosis. This research provides a more detailed explanation of the role of the JAK/STAT signaling pathway in malignant tumor fibrosis (Grohmann et al., 2018).

#### 2.3.3 Wnt/β-catenin signal transduction pathway

Wnt proteins bind to cell surface receptors, activating β-catenin, leading to its accumulation and translocation to the nucleus, where it regulates the expression of fibrosis-related genes. These genes generally pertain to ECM synthesis and fibroblast activation (Feng et al., 2018). In lung adenocarcinoma, smoking induces the downregulation of filamin A interacting protein 1-like (FILIP1L), which activates the Wnt/β-catenin signaling pathway, resulting in mucin secretion, inflammation, and fibrosis (Kwon et al., 2022). In oral submucous fibrosis and OSCC tissues, hypermethylation of dickkopf-1 may lead to its downregulation, causing abnormal activation of the Wnt/β-catenin signaling pathway, potentially playing a crucial role in the pathogenesis of oral submucous fibrosis (He et al., 2020). Additionally, proteins associated with the Wnt/β-catenin pathway are highly expressed in pancreatic exocrine tissues, with significant alterations in their cellular and subcellular expression patterns, correlating with increased fibrosis (Bläuer et al., 2019). Stearoyl-CoA desaturase (SCD) in liver tumor-initiating stem-like cells (TIC) is regulated by Wnt/β-catenin signaling. The monounsaturated fatty acids produced by SCD stabilize LRP5/6 mRNA, forming a positive feedback loop that amplifies Wnt signaling, which in turn promotes liver fibrosis and tumor growth (Lai et al., 2017).

#### 2.3.4 Notch signal transduction pathway

**FIGURE 1:**
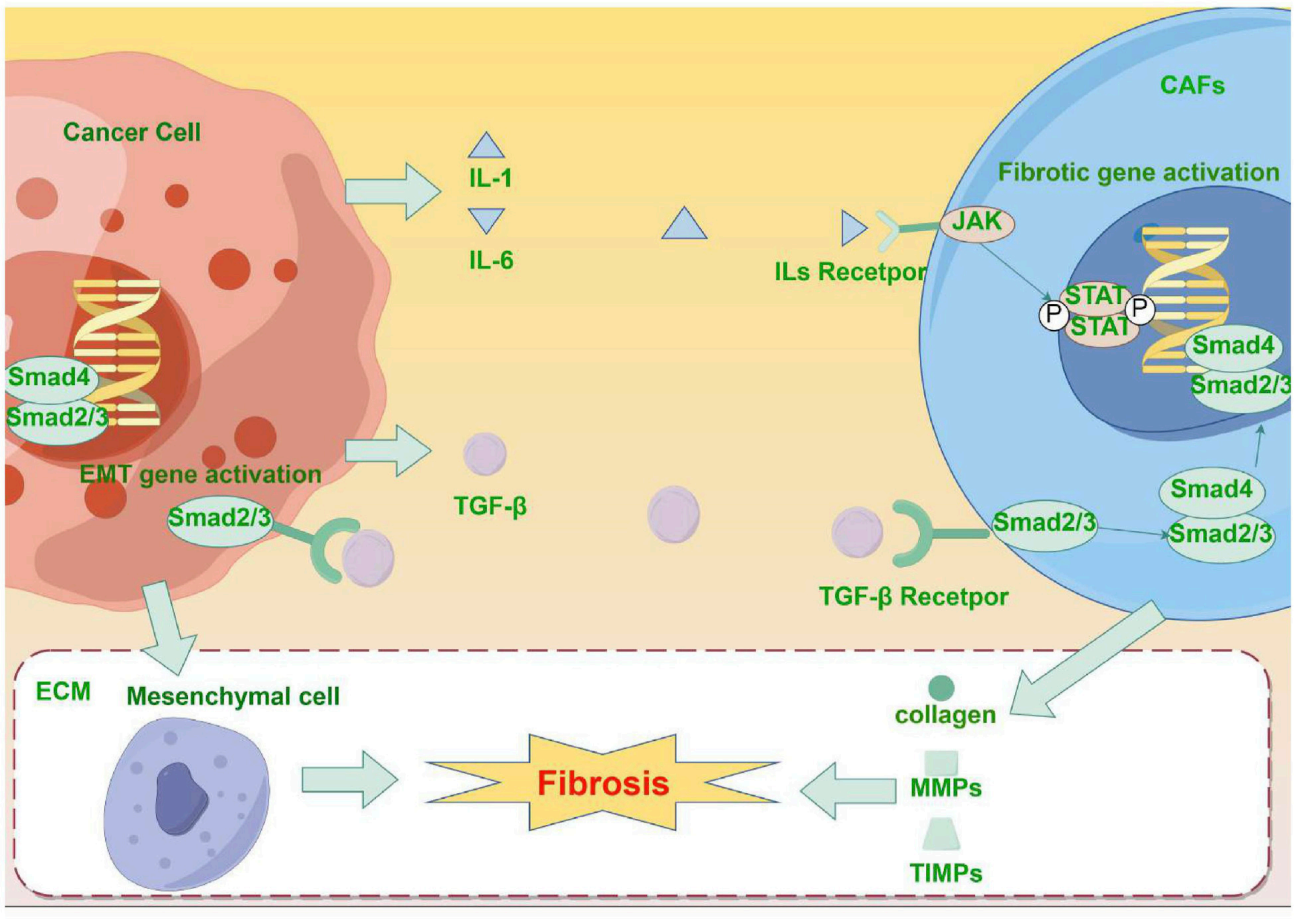
(By Figdraw, ID: RASTR41933) Mechanism of fibrosis promotion by malignant tumors: Tumor cells secrete pro-fibrotic factor TGF-β and activate CAFs through the TGF-β/Smad signaling pathway. Furthermore, tumor cells and CAFs secrete inflammatory factors (such as IL-1 and IL-6), which can further induce the production of additional pro-fibrotic factors, thus further activating CAFs. The activated CAFs promote the synthesis of collagen, fibronectin, and other ECM components, and affect ECM degradation and remodeling by regulating the secretion of MMPs and TIMPs. Additionally, the activation of the TGF-β/Smad signaling pathway is linked to the occurrence of EMT in malignant tumors, and the interaction between EMT and ECM remodeling further accelerates the fibrosis process.

The Notch signaling pathway is activated by the interaction between Notch receptors and ligands. After receptor activation, proteolytic cleavage releases the Notch intracellular domain (NICD), which then translocates to the nucleus to regulate the transcription of specific genes. Suppressing Notch signaling can inhibit the activation of the classical TGF-β1 pathway and reduce the peritumoral desmoplastic reaction in cholangiocarcinoma (Mancarella et al., 2022). In liver cancer cells chronically exposed to low concentrations of cadmium, the activation of Notch and AKT/mTOR signaling pathways can induce the expression of the pro-inflammatory cytokine tumor necrosis factor-α (TNF-α) and its downstream target TNF-α-induced protein 8 (TNFAIP8), thus regulating fibrosis and oncogenic signaling in liver cancer cells (Niture et al., 2023). Refer to Figure 1 for the mechanisms by which malignant tumors promote fibrosis.

#### 2.3.5 The cross-talk effects of signaling pathways

It is worth noting that during the fibrosis process in malignant tumors, multiple signaling pathways do not function independently but often co-regulate fibrosis and tumor progression through complex interaction mechanisms. The interactions between different signaling pathways form a highly integrated network, which has a profound impact on the tumor microenvironment, cell proliferation, invasion, and treatment resistance. For instance, TGF-β1-induced activation of activating transcription factor 4 (ATF4) is dependent on the activation of the classical TGF-β1/Smad3 signaling and mTORC1-4E-BP1. ATF4 then promotes the *de novo* synthesis of enzymes from the serine-glycine biosynthesis pathway and transcription of the GLUT1 gene. This process meets the biosynthetic demands required for enhanced ECM synthesis (Selvarajah et al., 2019).

## 3 The effect of fibrosis on tumor progression

### 3.1 Enhances tumor proliferation and survival

Fibrosis results in the abnormal accumulation of extracellular matrix (ECM), especially the increase in collagen, fibronectin, and hyaluronic acid. These ECM components not only offer structural support for tumor cells but also interact with cell surface receptors, activating signaling pathways that promote proliferation and survival. In liver cancer, Sema3C supports tumor fibrosis by promoting the proliferation of hepatic stellate cells (HSCs). Moreover, Sema3C interacts with NRP1 and ITGB1 receptors, activating the AKT/Gli1/c-Myc signaling pathway, promoting the self-renewal and proliferation of HCC cells (Peng et al., 2024). The increased tissue stiffness due to fibrosis further promotes tumor cell proliferation and survival via mechanotransduction pathways, such as the YAP pathway (Schrader et al., 2011; Deng et al., 2022).

Fibroblasts and CAFs within the fibrotic microenvironment secrete numerous growth factors, such as TGF-β and EGF. These factors facilitate tumor cell proliferation and survival by activating downstream signaling pathways. In prostate cancer, TGF-β1 is recognized as a highly secreted growth factor in CAFs, significantly enhancing tumor cell growth and proliferation in both *in vivo* and *in vitro* settings (Dy et al., 2019). In PDAC, CAF-derived thrombospondin 1 (TSP1) activates TGF-β signaling, leading to the loss of Smad4 expression in cancer cells and accelerating their proliferation and migration (Matsumura et al., 2022). In cholangiocarcinoma, CAF-secreted TSP-4 binds to integrin α2 on cancer cells, activating HSF1 and Akt signaling pathways. Activated HSF1 further enhances TGF-β1 expression and secretion, inducing the transformation of fibroblasts into CAFs and creating a positive feedback loop that promotes cell proliferation and advances cholangiocarcinoma progression (Shi et al., 2021).

### 3.2 Enhances angiogenesis

#### 3.2.1 Secretion of angiogenesis-promoting factors

In malignant tumors, fibrosis promotes angiogenesis through various mechanisms, supplying the necessary nutrients and oxygen for tumor growth and expansion. During the fibrosis process in malignant tumors, fibroblasts and CAFs are activated, leading to the secretion of significant amounts of pro-angiogenic factors, including vascular endothelial growth factor (VEGF), platelet-derived growth factor (PDGF), and basic fibroblast growth factor (bFGF). These factors interact with receptors on vascular endothelial cells, activating signaling pathways that promote the formation of new blood vessels (Sobierajska et al., 2020).

In several malignant tumors, such as head and neck squamous cell carcinoma, RCC, and cholangiocarcinoma, CAFs can directly secrete VEGF to promote angiogenesis (Sun et al., 2022; Zhou et al., 2022; Liu J. et al., 2023). In colorectal cancer patients, exosomes released by CAFs enhance endothelial cell proliferation, migration, and angiogenesis by increasing the expression and secretion of VEGF. Specifically, circ_0084043 is highly expressed in CAF-derived exosomes and regulates HIF-1α and VEGFA by sponging miR-140-3p, suggesting that the circ_0084043/miR-140-3p/VEGF signaling pathway plays a critical role in CAF exosome-induced angiogenesis (Payervand et al., 2024). miR-210 secreted by lung cancer cells enhances angiogenesis by increasing VEGF via the activation of the JAK2/STAT3 signaling pathway in CAFs (Fan et al., 2020). Similarly, research by Dai et al. (2022) showed that CAF-derived extracellular vesicles promote angiogenesis in colorectal adenocarcinoma cells via the miR-135b-5p/FOXO1 axis, indicating the crucial role of non-coding RNAs in enhancing the secretion of angiogenic factors by CAFs.

Moreover, several signaling pathways are also crucial in regulating VEGF secretion. When CAFs are co-cultured with glioma C6 cells, the expression levels of VEGF-A and EGF proteins are significantly elevated, thereby enhancing glioma cell invasiveness, proliferation, and angiogenesis (Zhang S. et al., 2023). In triple-negative breast cancer (TNBC), particularly in patients with BRCA1 mutations, iCAFs have been found to be enriched and promote angiogenesis by interacting with tumor endothelial cells (TECs) via VEGF signaling. iCAFs activate angiogenesis-related genes (such as FLT1 and KDR) in TECs through the VEGF signaling pathway, promoting endothelial cell migration and sprouting angiogenesis (Lee et al., 2024). In breast cancer, the upregulation of VEGF-A and IL-8, along with their upstream effectors mTOR and HIF-1α, can enhance the pro-angiogenic potential of CAFs (Al-Kharashi et al., 2022). In melanoma, CD38-positive CAFs promote tumor cell migration and invasion, as well as endothelial cell tube formation, by secreting factors like VEGF-A, FGF-2, and CXCL-12 through paracrine signaling *in vitro* (Ben Baruch et al., 2020).

PDGF and other angiogenesis-promoting factors likewise play a crucial role in driving angiogenesis facilitated by CAFs. Chu et al. (2022) discovered that VEGF, angiopoietin, bFGF, and other factors secreted by CAFs are crucial in the angiogenesis of precancerous and malignant lesions in laryngeal cancer. In OSCC, reprogramming of glucose metabolism results in increased secretion of angiogenesis-promoting factors (VEGF-A, PDGF-C, and MMP9) by CAFs, which enhances the angiogenic phenotype (Li X. et al., 2022). In cholangiocarcinoma, CAFs secrete stem cell factor (SCF), which recruits mast cells and stimulates them to release hyaluronic acid (HA) via the MRGPRX2-Gαq signaling pathway. These bile-induced MCs subsequently release PDGF-B, which further enhances angiogenesis in cholangiocarcinoma (Shi et al., 2024).

#### 3.2.2 ECM remodeling

Fibrosis caused by malignant tumors results in excessive extracellular matrix (ECM) deposition, offering a physical scaffold for new blood vessel formation and supporting vascular expansion within the dense matrix. In cholangiocarcinoma, the overexpression of PI3Kδ is closely related to stromal remodeling, manifesting as a thick ECM at the basement membrane and significant angiogenesis and lymphangiogenesis. The mechanism involves PI3Kδ promoting ECM remodeling via the TGFβ/Src/Notch signaling pathway, which in turn enhances angiogenesis (Bou Malham et al., 2023). In bladder cancer, the Sigma 1 receptor (Sig1R) can regulate crosstalk between the ECM and tumor cells, facilitating ECM-mediated cell proliferation and angiogenesis (Feng et al., 2023).

#### 3.2.3 Hypoxia and the activation of HIF-1α

Fibrosis increases the density of tumor tissue, limiting oxygen diffusion and creating a hypoxic microenvironment. Hypoxia-inducible factors (HIFs) become stabilized and activated in hypoxic conditions, enhancing the expression of pro-angiogenic genes like VEGF, which drives the formation of new blood vessels (Yehia et al., 2015; De Marco et al., 2022). Pancreatic cancer features excessive desmoplastic reaction and a hypoxic microenvironment within the solid tumor mass. Hypoxia induces the production of HIF-1, which not only enhances the migration of pancreatic stellate cells (PSCs) and the expression of type I collagen but also increases VEGF secretion, promoting angiogenesis (Masamune et al., 2008; N et al., 2016).

### 3.3 Enhances immune evasion

The dense ECM structure created by fibrosis obstructs immune cell infiltration, diminishing their tumor-killing capacity and aiding tumor cells in evading immune surveillance. In the fibrotic tumor microenvironment, tumor-associated macrophages (TAMs) initiate collagen synthesis via the TGF-β signaling pathway, causing tumor tissue stiffening and establishing a metabolic environment that impairs CD8^+^ T cell function. Macrophages engaged in collagen synthesis deplete arginine in the environment and produce proline and secrete ornithine, which further suppresses the antitumor response of CD8^+^ T cells. Therefore, fibrosis not only physically repels CD8^+^ T cells but also weakens the immune response against cancer by altering the metabolic environment (Tharp et al., 2024). In non-small cell lung cancer (NSCLC), significant fibrosis corresponds with reduced T cell infiltration, resulting in impaired immune surveillance. Fibrosis not only accelerates tumor progression but also reduces the number and function of dendritic cells and alters macrophage phenotypes, further intensifying immune suppression (Herzog et al., 2023). Liver fibrosis enhances tumor immune evasion in hepatocellular carcinoma, resulting in decreased CD8^+^ T cell infiltration and increased expression of the immune checkpoint molecule programmed death-ligand 1 (PD-L1). Specifically, Golgi membrane protein 1 (GOLM1) in fibrosis induces PD-L1 expression via the activation of the EGFR pathway, thereby suppressing antitumor immune responses (Ke et al., 2021). In the lung adenocarcinoma microenvironment, CAFs increase the expression of PD-L1 in tumor cells by secreting cytokines like CXCL2. High PD-L1 expression allows tumor cells to suppress CD8^+^ T cell activity in the immune system, facilitating immune evasion (Inoue et al., 2019). Regulatory T cells (Tregs) and CAFs interact to collaboratively enhance fibrosis and immune suppression. Specifically, IL-33 enhances Treg cell activity through the IL1RL1 signaling pathway, and these Tregs interact with CAFs via the AREG/EGFR axis, inducing CAFs into a pro-fibrotic and immunosuppressive state (Sun et al., 2023). However, it is important to note that fibrosis in malignant tumors can facilitate tumor immune evasion while also constraining tumor size expansion (Li et al., 2021).

## 4 Fibrosis enhances treatment resistance

### 4.1 Chemotherapy resistance

Chemotherapy is a treatment approach that employs chemical agents to kill cancer cells or inhibit their growth and division, commonly used in the treatment of various malignant tumors. However, chemoresistance is a significant challenge in the treatment of malignant tumors, and it is often accompanied by fibrosis in affected patients. In a pancreatic ductal adenocarcinoma model, ectopic tumors showed more pronounced fibrosis, which led to increased resistance to FOLFIRINOX chemotherapy. Despite similar drug absorption in tumor tissues, fibrosis and microenvironmental differences significantly impacted the treatment response (Erstad et al., 2018). In breast cancer, fibrosis-related signaling pathways are significantly upregulated in patients who do not achieve a complete response to neoadjuvant chemotherapy; patients with high fibrosis have lower complete response rates and shorter survival durations (Wang X. et al., 2024).

Fibrosis is frequently accompanied by epithelial-mesenchymal transition (EMT), which converts tumor cells from an epithelial phenotype to a mesenchymal phenotype. EMT provides tumor cells with enhanced migratory ability and resistance to apoptosis, thereby increasing their resistance to chemotherapy. In 5-Fu-resistant (5-FU) breast cancer cell lines, tumor cells induce normal dermal fibroblasts to convert into a CAF phenotype via TGF-β1 paracrine signaling, promoting fibrosis, reducing E-cadherin expression, and facilitating EMT (Chandra Jena et al., 2021). CAFs can transfer exosomes to colorectal cancer cells, promoting stemness and EMT in CRC cells, which in turn enhances resistance to 5-FU/oxaliplatin (L-OHP) chemotherapy. Mechanistically, exosomes induce miR-92a-3p production, activating the Wnt/β-catenin pathway, inhibiting FBXW7 and MOAP1 expression, and suppressing mitochondrial apoptosis, thereby enhancing stemness and chemoresistance (Hu et al., 2019). In ovarian cancer, CAFs may activate the Wnt/β-catenin pathway via the CXCL12/CXCR4 axis, promoting cisplatin resistance by inducing EMT (Zhang F. et al., 2020). IL-6 derived from CAFs plays a crucial role in maintaining the paracrine loop between CAFs and NSCLC cells by enhancing EMT in NSCLC cells. This paracrine loop enhances intercellular communication, which subsequently leads to the development of chemoresistance (Shintani et al., 2016).

In addition to EMT, fibrosis can promote chemoresistance by activating tumor stem cell properties and anti-apoptotic signaling pathways. In PDAC, proliferating resident macrophages (proliferating rMφs) significantly increase tumor resistance to chemotherapy by promoting fibrosis and immune suppression. Multi-omics analysis found that these macrophages promote cancer cell survival during chemotherapy by producing more deoxycytidine (dC) and less dC kinase (dCK), reducing the absorption of gemcitabine (Zhang J. et al., 2023). Additionally, CAFs promote tumor fibrosis via the IL1β-IRAK4 signaling pathway, which enhances tumor cell survival and proliferation, resulting in gemcitabine resistance (Zhang et al., 2018). In lung adenocarcinoma, cancer stem cells (CSCs) secrete the acute-phase protein serum amyloid A (SAA), remodeling the tumor microenvironment, promoting fibrosis, and enhancing cisplatin (DDP) chemoresistance (Wang et al., 2023). In ovarian cancer, high expression of CHI3L1 (a secretory glycoprotein) is closely linked to fibrosis. CHI3L1 activates the Akt and Erk signaling pathways, enhancing the expression of β-catenin and SOX2, promoting stem-like characteristics in ovarian cancer cells, such as resistance to apoptosis, thereby increasing paclitaxel chemoresistance (Lin et al., 2019). In CAF-derived exosomes, the significantly upregulated circBIRC6 promotes the SUMOylation of XRCC4, enhancing its interaction with SUMO1 at lysine 115, facilitating XRCC4 chromatin localization, and increasing pancreatic cancer cell resistance to oxaliplatin (Zheng et al., 2023). In conclusion, fibrosis can enhance chemotherapy resistance through multiple mechanisms, including EMT, CSC, and anti-apoptotic pathways.

### 4.2 Resistance to immunotherapy

Presently, immunotherapy is an emerging cancer treatment method that fights cancer by enhancing or regulating the immune system. Immune checkpoint inhibitors are widely used immunotherapy strategies, among which PD-1/PD-L1 inhibitors (like pembrolizumab and nivolumab) and CTLA-4 inhibitors (such as ipilimumab) have shown significant therapeutic potential in tumor immunotherapy (Li W. et al., 2022). In tumors that respond to immunotherapy, the TME shows enrichment of immune cells and CAFs, along with pro-inflammatory signaling and ECM remodeling, which aligns with proliferative fibrosis and immune-mediated tumor regression. However, tumor heterogeneity may result in immune-deficient regions, promoting immune evasion and early recurrence via HCC-CAF interactions and the expression of cancer stem cell markers. This indicates that fibrosis may contribute to immunotherapy resistance in certain cases, heightening treatment challenges (Zhang M. et al., 2023). In breast cancer, fibrosis facilitates immunotherapy resistance by increasing TAMs, EMT, fibroblast proliferation, ECM enhancement, and Wnt pathway activation. These alterations together create an immune-tolerant microenvironment, diminishing the effectiveness of PD-1 inhibitors (Yuan et al., 2022). Further research by Song et al. (2024) revealed a link between anti-PD-L1 therapy and fibrosis: During liver fibrosis, pathogenic Th17 cells (pTh17) significantly increase, and anti-PD-L1 therapy promotes pTh17 cell infiltration and activation in the liver. These pTh17 cells secrete IL-17A, which increases PD-L1 expression on the surface of hepatocellular carcinoma cells, further worsening liver cirrhosis and leading to resistance to anti-PD-L1 therapy (Song et al., 2024).

### 4.3 CAFs enhance resistance to targeted therapy

Targeted therapy is a form of cancer treatment that specifically targets certain molecules or signaling pathways in cancer cells. Unlike traditional chemotherapy, targeted therapy precisely identifies and inhibits abnormal proteins or genetic mutations in cancer cells, preventing tumor growth and spread while minimizing harm to normal cells.

#### 4.3.1 Resistance to tyrosine kinase inhibitors

In the fibrotic microenvironment, CAFs play a key role in promoting resistance to tyrosine kinase inhibitors (TKIs). For example, in RCC, CAFs facilitate resistance to VEGFR-TKIs (Ambrosetti et al., 2022). In HCC, bioinformatics analysis identified SPP1 secreted by CAFs as a candidate molecule for resistance to sorafenib and lenvatinib. CAF-secreted SPP1 activates the RAF/MAPK and PI3K/AKT/mTOR pathways via the integrin-PKCα signaling pathway and promotes EMT, resulting in TKI resistance (Eun et al., 2023). CAFs enhance the secretion of HGF and IGF-1, activating the c-met and IGF-1R receptors, leading to increased ANXA2 expression and phosphorylation, inducing EMT and resulting in resistance to EGFR-TKIs (e.g., gefitinib) in NSCLC (Yi et al., 2018). Similarly, in NSCLC, CAFs derived from osimertinib-resistant cells secrete higher levels of IL-6, IL-8, and hepatocyte growth factor (HGF), express stronger CAF markers such as α-SMA, FAP, and PDGFR, and increase stemness and osimertinib resistance in NSCLC cells (Huang W. et al., 2021). In EGFR-TKI-resistant tumors, part of the CAF-derived tumor stroma is composed of EMT-derived tumor cells that express resistance markers, such as epithelial membrane protein-1. CAFs secrete paracrine factors that reduce the inhibitory effects of TKIs on pEGFR and pMAPK, thereby promoting tumor cell survival and drug resistance (Sr et al., 2010). In gastric cancer, cancer cells secrete lactate, inducing CAFs to produce BDNF, activating the TrkB-Nrf2 signaling pathway, inhibiting anlotinib-induced apoptosis and reactive oxygen species (ROS) generation, thus reducing drug efficacy (Jin et al., 2021). In RCC, CAFs increase sunitinib resistance by secreting CXCL3, which activates the CXCR2-ERK1/2 signaling pathway in tumor cells, promoting EMT and stemness (Wang Y. et al., 2024).

#### 4.3.2 Resistance to monoclonal antibodies

Monoclonal antibodies (mAbs) are a crucial class of drugs in targeted therapy, specifically targeting certain antigens or receptors on cancer cell surfaces. They kill specific cancer cells by directly blocking signal transduction, activating ADCC, or inducing complement-dependent cytotoxicity (CDC). Common monoclonal antibodies include trastuzumab, bevacizumab, and cetuximab.

Trastuzumab can target the HER2 receptor and is used for treating HER2-positive breast cancer and gastric cancer. CAFs are enriched in trastuzumab-resistant HER2-positive breast cancer cases. These CAFs secrete immunosuppressive factors IDO1 and TDO2, inhibiting NK cell-mediated antibody-dependent cellular cytotoxicity (ADCC), thereby causing resistance to trastuzumab (Du et al., 2023). CAF-derived Neuregulin 1 (NRG1) also mediates trastuzumab resistance in breast cancer by activating the HER3/AKT signaling pathway. However, pertuzumab may reverse resistance by targeting this pathway (Guardia et al., 2021). Mao et al. (2015)’s research shows that CAFs can induce trastuzumab resistance by expanding cancer stem cells and activating multiple pathways including NF-κB, JAK/STAT3, and PI3K/AKT.

Bevacizumab targets VEGF to inhibit angiogenesis and is used in the treatment of colorectal cancer, non-small cell lung cancer, renal cell carcinoma, and more. In OSCC, CAFs play a key role in angiogenesis by secreting sEVs. CAF-derived sEVs bind to VEGF and activate the VEGFR2 signaling pathway in human umbilical vein endothelial cells (HUVECs). Even after Bevacizumab treatment, VEGF bound to sEVs can continue to activate VEGFR2. This indicates that sEVs secreted by CAFs can bind VEGF via heparan sulfate proteoglycans on their surface, making them resistant to Bevacizumab (Li et al., 2020).

**FIGURE 2:**
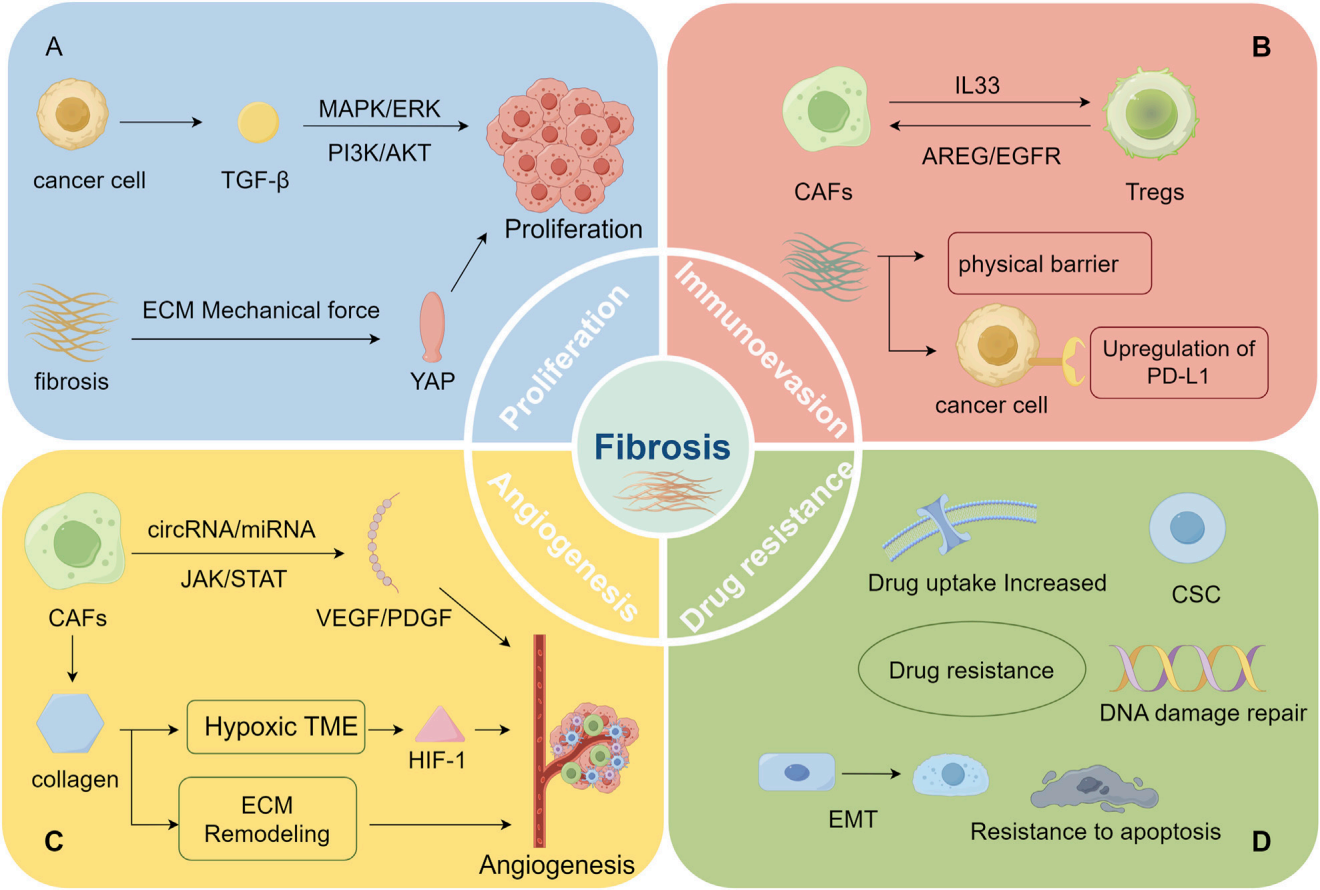
(By Figdraw, ID: RYWAR2b2b6) Fibrosis facilitates malignant tumor progression: **(A)** enhances tumor proliferation, **(B)** promotes immune evasion, **(C)** drives angiogenesis, **(D)** increases treatment resistance **(A)** Malignant tumors enhance tumor cell proliferation by secreting TGF-β, which activates pathways such as PI3K-AKT and MAPK/ERK. Furthermore, the increased tissue stiffness from fibrosis promotes tumor cell proliferation via mechanotransduction pathways (like the YAP pathway). **(B)** The interaction between Treg cells and CAFs not only promotes fibrosis but also intensifies immunosuppression. Moreover, the dense ECM structure resulting from fibrosis obstructs immune cell infiltration and aids tumor cells in evading immune surveillance by upregulating PD-L1 expression. **(C)** CAFs enhance the secretion of pro-angiogenic factors like VEGF and PDGF via the regulation of non-coding RNAs and signaling pathways such as JAK, thereby promoting tumor angiogenesis. The excessive deposition of ECM provides a physical scaffold for new blood vessel formation, supporting vascular expansion within the dense matrix. Additionally, excessive ECM accumulation restricts oxygen, activating HIF-1 and further promoting angiogenesis. **(D)** Fibrosis facilitates treatment resistance in malignant tumors through multiple mechanisms, including CSC activation, EMT induction, apoptosis inhibition, enhanced DNA damage repair, and reduced drug uptake, thereby weakening the efficacy of chemotherapy, targeted therapy, and immunotherapy.

Cetuximab targets EGFR and is used for targeted therapy in colorectal cancer and head and neck squamous cell carcinoma (HNSCC). In CRC, CAFs significantly increase CRC cell resistance to Cetuximab by regulating the expression of the EMT key factor SNAI1 and remodeling the ECM (Galindo-Pumariño et al., 2022). In HNSCC, TGF-β-activated CAFs limit Cetuximab efficacy by upregulating the TGF-β signaling pathway, thereby enhancing drug resistance in the tumor microenvironment (Yegodayev et al., 2020). Further studies indicate that CAF-derived MMP-1 expression increases in both tumor cells and CAFs, promoting resistance to Cetuximab (Johansson et al., 2012). Refer to Figure 2 for fibrosis-promoted malignant tumor progression and treatment resistance. Refer to Table 1 for details on how fibrosis promotes treatment resistance.

**TABLE 1:** Summarize the mechanisms and associated signaling pathways through which fibrosis promotes resistance to chemotherapy, immunotherapy, and targeted therapy.

Type of drug	Drug	Disease	Mechanism of resistance	Signaling pathway	Reference
Chemotherapy	5-Fu/L-OHP	CRC	EMT, CSC	miR-92a-3p/Wnt/β-catenin	Hu et al. (2019)
	5-Fu	Breast cancer	EMT	--	Chandra Jena et al. (2021)
	Gemcitabine	PDAC	Drug uptake Increased	--	Zhang J. et al. (2023)
	Gemcitabine	PDAC	Resistance to apoptosis	IL1β-IRAK4	Zhang et al. (2018)
	DDP	Ovarian cancer	EMT	CXCL12/CXCR4-Wnt/β-catenin	Zhang F. et al. (2020)
	DDP	Lung adenocarcinoma	CSC	--	Wang et al. (2023)
	Paclitaxel	Ovarian cancer	CSC	CHI3L1/Akt/Erk-β- catenin	Lin et al. (2019)
	L-OHP	PDAC	DNA damage repair	circBIRC6-XRCC4	Zheng et al. (2023)
ICI	PD-1 mAb	Breast cancer	EMT, TAM increase	Wnt signaling pathway	Yuan et al. (2022)
TKI	Sorafenib/lenvatinib	HCC	EMT\	RAF/MAPK、PI3K/AKT/mTOR	Eun et al. (2023)
	Gefitinib	NSCLC	EMT	HGF/IGF-1/c-met、 IGF-1R-ANXA2	Yi et al. (2018)
	Axitinib	NSCLC	CSC	--	Huang W. et al. (2021)
	Sunitinib	RCC	CSC, EMT	CXCR2-ERK1/2	Wang Y. et al. (2024)
	Anlotinib	Gastric cancer	Resistance to apoptosis and ROS	BDNF-TrkB-Nrf2	Jin et al. (2021)
mAbs	Trastuzumab	Gastric cancer, Breast cancer	Resistance to ADCC	--	Du et al. (2023)
	Trastuzumab	Breast cancer	Resistance to apoptosis	NRG1/HER3/AKT	Guardia et al. (2021)
	Trastuzumab	Breast cancer	CSC	NF-κB, PI3K/AKT and JAK/STAT3	Mao et al. (2015)
	Bevacizumab	OSCC	Continuous activation of VEGF	--	Li et al. (2020)
	Cetuximab	HNSCC, CRC	EMT, ECM Remodeling	--	Galindo-Pumariño et al. (2022)
	Cetuximab	HNSCC	Upregulation of the TGF-β signaling pathway	TGF-β signaling pathway	Johansson et al. (2012)

5-Fu, 5-Fluorouracil; L-OHP, Oxaliplatin; CRC, Colorectal cancer; EMT, Epithelial–mesenchymal transition; CSC, Cancer stem cell; PDAC, Pancreatic Ductal Adenocarcinoma; DDP, Cisplatin; TAM, Tumor-associated macrophages; HCC, Hepatocellular carcinoma; NSCLC, Non-small cell lung cancer; RCC, Renal cell carcinoma; ADCC, Antibody dependent cell-mediated cytotoxicity; OSCC, Oral squamous cell carcinoma; HNSCC, Head and neck squamous cell carcinoma.

## 5 Tumor therapeutic strategies targeting fibrosis

### 5.1 Nintedanib

#### 5.1.1 Clinical trials

Nintedanib is a small-molecule TKI with antifibrotic and anti-inflammatory properties, mainly used in the treatment of idiopathic pulmonary fibrosis. The clinical use of antifibrotic drugs such as Nintedanib can significantly improve the survival time of patients with certain malignant tumors. In refractory metastatic CRC, Nintedanib combined with capecitabine is well tolerated and clinically more effective than regorafenib or trifluridine/tipiracil monotherapy. In a study of 36 patients, the median progression-free survival (PFS) was 3.4 months, and the median overall survival (OS) was 8.9 months after 18 weeks (Boland et al., 2024). Nintedanib combined with chemotherapy significantly improved PFS in NSCLC patients, though it had no significant impact on OS. A meta-analysis of three randomized controlled trials involving 2,270 patients showed that PFS in the Nintedanib group was significantly better than in the placebo group (HR = 0.79; 95% CI 0.71–0.88, *p* < 0.0001) (Alhadeethi et al., 2024). Additionally, a multicenter retrospective study indicated that Nintedanib combined with docetaxel had some efficacy in advanced NSCLC patients following the failure of immune checkpoint inhibitors (ICI) and/or chemotherapy. In 96 patients, the objective response rate (ORR) was 18.8%, the disease control rate (DCR) was 57.3%, the median PFS was 3.0 months, and the median OS was 8.0 months. Particularly in patients treated with Nintedanib and docetaxel after first-line ChT-ICI therapy, the ORR was 29.2%, the DCR was 66.7%, and the median PFS was 4.0 months (Ljubicic et al., 2023). These studies indicate that Nintedanib can effectively improve survival time in patients with certain malignant tumors.

However, there are ongoing debates regarding the response rate and safety of Nintedanib. In a double-blind, randomized, phase 2 trial adding Nintedanib to neoadjuvant chemotherapy for muscle-invasive bladder cancer, the pathological complete response rate (pCR) was similar between the Nintedanib and placebo groups (37% vs. 32%). However, the Nintedanib group showed a higher incidence of grade 3 or higher toxic events (93% vs. 79%), with the most common serious adverse events being thromboembolic events (30% vs. 21%) and neutropenia (39% vs. 11%) (Hussain et al., 2022). In ovarian cancer, the Nintedanib treatment group showed worse PFS and OS compared to the placebo group, along with higher toxicity (92% vs. 69% for grade 3/4 adverse events), primarily consisting of hematologic and gastrointestinal side effects (Ferron et al., 2023). Refer to Table 2 for the clinical trial results of Nintedanib.

**TABLE 2:** Efficacy and adverse reactions of Nintedanib in different malignant tumors.

Disease	Trial-registration	Phase	Case	OS (month)	PFS (month)	PFS HR (95% CI)	Serious treatment-related adverse events (Grade 3–4)	Reference
RAIR DTC	NCT01788982	II	56	--	3.7	0.65 (0.42–0.99)	50%	Leboulleux et al. (2024)
MTC	NCT01788982	II	20	--	7.0	0.49 (0.16–1.53)	59.1%	Leboulleux et al. (2024)
NSCLC	NCT02299141	--	20	11.3	4.3	--	35%	Auberle et al. (2024)
CRC	NCT02393755	I/II	42	8.9	3.4	--	44%	Boland et al. (2024)
SCLC	jRCTs031190119	II	33	13.4	4.2	--	81.8%	Ikeda et al. (2024)
NSCLC	--	--	27	15.8	5.4	--	44.4%	Makiguchi et al. (2023)
Ovarian Cancer	NCT01583322	II	188	37.7	14.4	1.50	96%	Ferron et al. (2023)
NSCLC	jRCTs071180049	III	243	15.3	6.2	0.68 (0.50–0.92)	72.5%	Otsubo et al. (2022)
NSCLC	--	II	59	6.9	2.7	--	53.7%	Auliac et al. (2021)
CRC	NCT01362361	II	53	17.1	8.1	0.65 (0.32–1.30)	73.1%	Ettrich et al. (2021)

PFS, Progression-Free-Survival; OS, Overall survival; HR, Hazard Ratio; RAIR DTC, Radioiodine-refractory differentiated thyroid cancer; MTC, Medullary thyroid cancer.

#### 5.1.2 Sensitization to chemotherapy and immunotherapy

Combining Nintedanib with immunotherapy or chemotherapy drugs can significantly improve treatment outcomes and promote tumor cell death. Nintedanib significantly inhibits tumor growth in mouse models. When combined with anti-PD-1 antibodies, Nintedanib enhances antitumor efficacy primarily by reducing the number of TAMs and polarizing them into the antitumor M1 phenotype. The combination therapy also restores macrophage phagocytic function, enhancing treatment effectiveness (Tada et al., 2023). In malignant tumors, combining Nintedanib with PD-L1 enhances immune cell infiltration and activation within the tumor, boosts interferon-γ response, and activates MHC class I-mediated antigen presentation. It also promotes PD-L1 expression and STAT3 phosphorylation, thereby improving the effectiveness of immunotherapy (Tu et al., 2022). In PDACs, Nintedanib inhibits CAF secretion of IL-6 by blocking the PDGFRβ signaling pathway. Moreover, MSLN-targeted chimeric antigen receptor-NK cells combined with Nintedanib significantly enhanced tumor-killing ability in xenograft models, triggering robust NK cell infiltration (Lee et al., 2023).

In a xenograft model derived from gastric adenocarcinoma cells, Nintedanib inhibited tumor cell proliferation, reduced tumor angiogenesis, and increased tumor cell death. Notably, when combined with docetaxel and irinotecan, it significantly extended the animals’ survival (Awasthi et al., 2023).

### 5.2 Pirfenidone (PFD)

#### 5.2.1 PFD suppresses tumor invasion capability

Clinically, PFD is an approved drug used to treat idiopathic pulmonary fibrosis. It alleviates fibrotic responses by inhibiting TGF-β and other profibrotic factors and can significantly reduce tumor invasiveness by inhibiting EMT, regulating immune responses in the tumor microenvironment, and remodeling the ECM. For instance, PFD can inhibit the growth of breast tumors in mice and alcohol-promoted metastasis (Li H. et al., 2024). In TNBC, PFD reduces the expression of EMT-related transcription factors and mesenchymal genes by inhibiting the TGF-β/Smad signaling pathway, thereby inhibiting the proliferation, migration, and invasion of breast cancer cells while promoting apoptosis (Luo et al., 2023). PFD promotes the downregulation of ZEB1 via miR-200 in NSCLC exosomes, slowing down migration, invasion, and EMT processes (Liu et al., 2022). In RCC, PFD significantly inhibits the progression of renal cancer by targeting the TGF-β signaling pathway. PFD decreases TGF-β expression and secretion, blocking TGF-β-induced EMT and thus reducing the proliferation, migration, and invasion of renal cancer cells. Additionally, PFD enhances the immunosuppressive tumor microenvironment by limiting the recruitment of tumor-infiltrating myeloid-derived suppressor cells (MDSCs) (Wang et al., 2022). PFD targets CAFs, inhibiting EMT and stemness features in breast cancer cells. In breast cancer samples with a high stromal index, CAFs promote cancer cell spheroid formation and induce the expression of YAP1, VIM, and CD44. PFD treatment significantly reduces cancer cell migration and the protein expression levels of these genes (Es et al., 2021). PFD inhibits the expression of CAFs, hyaluronic acid, and collagen I, reducing tumor stromal pressure, eliminating the immunosuppressive microenvironment, and increasing cytotoxic T lymphocyte infiltration, thereby remodeling the desmoplastic tumor microenvironment. Moreover, PFD, in combination with therapies targeting the mitochondrial ROS-PYK2 pathway, significantly inhibits the growth and metastasis of malignant breast cancer (Zuo et al., 2021). PFD effectively eliminates the ethanol-mediated promotion of the TGF-β/RUNX3/Snail axis in CRC metastasis by specifically blocking the TGF-β signaling pathway (Zheng et al., 2019).

#### 5.2.2 Sensitization to chemotherapy and immunotherapy

In chemotherapy, PFD can significantly enhance tumor cell death. PFD can reprogram several biological pathways, inhibiting tumor cell secretion of PDGF by downregulating the TGM2/NF-kB/PDGFB pathway, thus exerting antifibrotic effects. This leads to a reduction in collagen X and fibronectin secretion by CAFs, and in a mouse pancreatic tumor orthotopic model, PFD showed the potential to enhance gemcitabine sensitivity (Lei et al., 2024). PFD’s use in NSCLC primarily focuses on its antitumor and chemosensitizing effects. PFD exerts anticancer effects by inhibiting the TGF-β1 signaling pathway, reducing lactate and ATP production, and thus inhibiting glycolysis. When combined with cisplatin, PFD enhances the targeted inhibition of TGF-β1, improving chemotherapy sensitivity in A549 and H1299 cells (Zhang S. et al., 2024). In TNBC, PFD inhibits the TGF-β/Smad signaling pathway, reducing the expression of EMT-related transcription factors and mesenchymal genes, inhibiting breast cancer cell proliferation, migration, and invasion, and promoting apoptosis. Additionally, although PFD has a relatively mild standalone antitumor effect *in vivo*, its combination with nab-paclitaxel (nab-PTX) significantly enhances the anticancer effect in TNBC (Luo et al., 2023).

PFD demonstrates significant potential when combined with immunotherapy. When combined with PD-L1 inhibitors, PFD significantly delays tumor growth, improves survival rates, enhances both innate and adaptive immune responses, increases immune cell infiltration, and optimizes T cell localization. This combination therapy also effectively alleviates lung fibrosis and reduces tumor growth (Qin et al., 2020). In bladder cancer, the combination of PD-L1 inhibitors and PFD can significantly inhibit bladder cancer progression, potentially by modulating the tumor immune microenvironment and inhibiting tumor cell epithelial-mesenchymal transition (Chen et al., 2024).

#### 5.2.3 Targeted drug delivery increases therapeutic efficacy

In pancreatic cancer, PFD combined with miR-138-5p, delivered through targeted engineered exosomes, successfully reprogrammed CAFs, inhibiting their pro-tumor effects. The combination inhibited the TGF-β signaling pathway and collagen synthesis, significantly improving the TME, reducing tumor pressure, enhancing the penetration of the chemotherapeutic drug gemcitabine, and increasing the sensitivity of cancer cells to chemotherapy (Zhou et al., 2024). In Jia et al. (2024)’s study, cell membrane-fused liposomes were used for targeted delivery of PFD and doxorubicin to inhibit CAF activity and remodel the TME, thereby significantly enhancing chemotherapy efficacy in TNBC. Furthermore, the optimized delivery strategy amplified the effects of anti-PD-L1 immunotherapy (Jia et al., 2024). Targeted drug delivery provides new insights for precision medicine in clinical practice.

### 5.3 Galunisertib

#### 5.3.1 Clinical trials

Galunisertib is a selective inhibitor of TGF-β receptor type I (ALK5), capable of blocking TGF-β signaling, inhibiting tumor growth and metastasis, and demonstrating potential in the treatment of malignant tumors. In a trial evaluating Galunisertib combined with nivolumab for NSCLC treatment, patients received Galunisertib (150 mg, 14 days on/14 days off) along with nivolumab (3 mg/kg IV every 2 weeks). 24% of patients showed confirmed partial responses, and 16% of patients exhibited stable disease. The median progression-free survival was 5.26 months, and the median overall survival was 11.99 months. The response rate for locally advanced NSCLC is generally low, about 10%–20%, suggesting that this drug may partially increase patient survival rates (Nadal et al., 2023). In another study, patients with locally advanced rectal cancer received Galunisertib-containing neoadjuvant chemoradiotherapy, resulting in an increase in the complete response rate to 32% with good tolerability, markedly increases the complete response rate compared to the previous treatment regimen (Yamazaki et al., 2022). Galunisertib has demonstrated potential in increasing complete response rates clinically, and its efficacy deserves further evaluation in randomized trials.

#### 5.3.2 Sensitization to chemotherapy and immunotherapy

Fibrosis forms a physical barrier and also creates an immunosuppressive microenvironment by secreting multiple cytokines. Anti-TGF-β drugs reduce fibrosis and can partially relieve this immunosuppression, promoting the infiltration of T cells and other immune effector cells into the tumor area. In OSCC, Galunisertib downregulates TGF-β signaling, enhances CD8^+^ T cell activity, and improves the efficacy of anti-PD-1 immunotherapy (Tao et al., 2024). In aggressive B-cell non-Hodgkin lymphoma (B-NHL), Galunisertib promotes immune system activation, reduces detrimental Treg cells, and prevents CD8^+^ T cell exhaustion (Rej et al., 2023). In PDAC, Galunisertib combined with dual immune checkpoint inhibitors (anti-PD-L1 and CTLA-4) significantly inhibits tumor growth and induces the infiltration of antitumor M1 macrophages. Additionally, it can enhance the immune system’s tumor-attacking ability by reducing the number of tumor-associated immunosuppressive cells (Rana et al., 2022). Galunisertib combined with IL-15-activated dendritic cells significantly enhances immunotherapy efficacy in highly invasive and metastatic mouse lymphoma. This combination therapy improves prognosis by inhibiting Treg cells in tumor-draining lymph nodes and spleen and through the inactivation of p-SMAD2 and Neuropilin-1 (Hira et al., 2020).

Galunisertib, when combined with chemotherapy drugs, enhances therapeutic efficacy. In B-NHL, Galunisertib enhances the antiproliferative and pro-apoptotic effects of doxorubicin and further inhibits tumor growth by upregulating p-P38 MAPK and inhibiting the TGF-β/Smad2/3 and PI3K/AKT signaling pathways (Rej et al., 2023).

### 5.4 Tranilast

Tranilast is an anti-allergic medication originally used to treat allergic conditions such as bronchial asthma, allergic rhinitis, and eczema. However, as research has advanced, Tranilast has demonstrated potential in the treatment of fibrosis-related diseases and certain cancers by inhibiting fibroblast activation, reducing malignant tumor resistance, and decreasing tumor proliferation. For instance, in CRC, Tranilast inhibits tumor growth by reducing tumor size, fibrosis, and angiogenesis. When combined with 5-FU, Tranilast further enhances the antitumor effect, leading to increased ROS production, decreased collagen deposition, and enhanced tumor necrosis (Hashemzehi et al., 2021).

#### 5.4.1 Tranilast impacts CAF function

Tranilast inhibits the migration of M2 macrophages by suppressing CXCL12 secretion by CAFs, while also inhibiting tumor growth, fibrosis, and the infiltration of M2 macrophages and mast cells. Additionally, it significantly promotes CD8^+^ lymphocyte infiltration into the tumor, thereby inducing cancer cell apoptosis via immune response (Nakamura et al., 2022). In NSCLC, Tranilast inhibits IL-6 secretion by CAFs, blocks CAF-induced upregulation of the STAT3 signaling pathway, reduces EMT, and reverses CAF-mediated resistance of NSCLC to osimertinib/selumetinib (Ochi et al., 2022). Furthermore, Tranilast, by inhibiting CAF activity, prevents them from promoting the survival and radioresistance of nasopharyngeal carcinoma cells via the IL-8/NF-κB pathway following radiotherapy (Huang W.-C. et al., 2021).

#### 5.4.2 Tranilast suppresses the TGF-β signaling pathway

In lung cancer, Tranilast inhibits TGF-β1-induced EMT and cell invasion by suppressing Smad4 expression, leading to reduced pleural dissemination of cancer cells (Takahashi et al., 2020). In breast cancer, Tranilast modulates the TGF-β signaling pathway by increasing AKT1 phosphorylation and reducing ERK1/2 phosphorylation, causing cell cycle arrest after the G1/S phase. Additionally, Tranilast upregulates p53, induces PARP cleavage, promotes tumor cell apoptosis, and modulates cell migration and invasion by inhibiting TGF-β (Subramaniam et al., 2010).

#### 5.4.3 Tranilast enhances the TME

Tranilast combined with Doxil treatment normalizes the TNBC TME by significantly reducing ECM components, increasing tumor blood vessel diameter and pericyte coverage, and improving tumor perfusion and oxygenation. These changes enhanced the antitumor immune response and improved therapeutic efficacy. Additionally, Tranilast restored T cell infiltration and reduced the migration of T cells away from immunosuppressive CAFs. The combination of Tranilast and Doxil also significantly increased the levels of immunostimulatory M1 macrophages in tumor tissue, enhancing the efficacy of immune checkpoint inhibitors (such as anti-PD-1/anti-CTLA-4) (Panagi et al., 2020).

## 6 Conclusion and outlook

In conclusion, as research deepens, the interaction between fibrosis and malignant tumors has received increasing attention. Fibrosis is not merely a consequence of tumor development but plays a crucial role in tumor progression, resistance to therapy, and immune evasion. Fibrosis facilitates tumor cell proliferation, migration, and invasion by altering the composition and structure of the extracellular matrix, while also offering a protective niche for tumor cells to evade immune surveillance. Additionally, fibrosis is closely linked to the activation of tumor-associated fibroblasts, which secrete various cytokines and growth factors, further exacerbating the malignancy of tumors.

Strategies targeting fibrosis in tumor treatment exhibit broad prospects, as fibrosis plays a key role in tumor progression and drug resistance. Inhibiting fibrosis-related signaling pathways, such as TGF-β, can not only suppress tumor cell proliferation and metastasis but also enhance the effects of chemotherapy, targeted therapy, and immunotherapy. For instance, TGF-β inhibitors can decrease fibrosis, improve drug permeability, and increase treatment effectiveness. Moreover, drugs targeting CAFs in the tumor microenvironment have demonstrated potential in preclinical research (Conte, 2022).

In the future, anti-fibrosis therapies are likely to become a crucial part of cancer treatment, especially when combined with current therapies, providing new options for patients with difficult-to-treat tumors. Through ongoing research, anti-fibrosis strategies will offer crucial support in enhancing treatment outcomes and improving patients’ quality of life.
